# Cardiovascular disease risk exacerbates brain aging among Hispanic/Latino adults in the SOL-INCA-MRI Study

**DOI:** 10.3389/fnagi.2024.1390200

**Published:** 2024-05-08

**Authors:** Ariana M. Stickel, Wassim Tarraf, Kevin A. Gonzalez, Alejandra Morlett Paredes, Donglin Zeng, Jianwen Cai, Carmen R. Isasi, Robert Kaplan, Richard B. Lipton, Martha L. Daviglus, Fernando D. Testai, Melissa Lamar, Linda C. Gallo, Gregory A. Talavera, Marc D. Gellman, Alberto R. Ramos, Vladimir Ivanovic, Stephan Seiler, Hector M. González, Charles DeCarli

**Affiliations:** ^1^Department of Psychology, San Diego State University, San Diego, CA, United States; ^2^Department of Healthcare Sciences, Institute of Gerontology, Wayne State University, Detroit, MI, United States; ^3^Department of Neurosciences, University of California, San Diego, La Jolla, CA, United States; ^4^Department of Biostatistics, University of North Carolina at Chapel Hill, Chapel Hill, NC, United States; ^5^Department of Epidemiology & Population Health, Albert Einstein College of Medicine, Bronx, NY, United States; ^6^Department of Neurology, Albert Einstein College of Medicine, Bronx, NY, United States; ^7^Institute for Minority Health Research, University of Illinois at Chicago, College of Medicine, Chicago, IL, United States; ^8^Department of Neurology & Neurorehabilitation, College of Medicine, University of Illinois at Chicago, Chicago, IL, United States; ^9^Rush Alzheimer’s Disease Research Center, Rush University Medical Center, Chicago, IL, United States; ^10^Department of Psychology, University of Miami, Miami, FL, United States; ^11^Department of Neurology, University of Miami Miller School of Medicine, Miami, FL, United States; ^12^Department of Radiology, Medical College of Wisconsin, Milwaukee, WI, United States; ^13^Department of Neurology, Klinikum Klagenfurt, Klagenfurt, Austria; ^14^Department of Neurology, University of California at Davis, Davis, CA, United States

**Keywords:** Hispanic/Latino heritage, cardiovascular disease risk, brain aging, brain volumes, infarcts

## Abstract

**Background:**

Cardiovascular disease (CVD) risk factors are highly prevalent among Hispanic/Latino adults, while the prevalence of MRI infarcts is not well-documented. We, therefore, sought to examine the relationships between CVD risk factors and infarcts with brain structure among Hispanic/Latino individuals.

**Methods:**

Participants included 1,886 Hispanic/Latino adults (50–85 years) who underwent magnetic resonance imaging (MRI) as part of the Study of Latinos—Investigation of Neurocognitive Aging-MRI (SOL-INCA-MRI) study. CVD risk was measured approximately 10.5 years before MRI using the Framingham cardiovascular risk score, a measure of 10-year CVD risk (low (<10%), medium (10- < 20%), and high (≥20%)). MR infarcts were determined as present or absent. Outcomes included total brain, cerebral and lobar cortical gray matter, hippocampal, lateral ventricle, and total white matter hyperintensity (WMH) volumes. Linear regression models tested associations between CVD risk and infarct with MRI outcomes and for modifications by age and sex.

**Results:**

Sixty percent of participants were at medium or high CVD risk. Medium and high CVD risk were associated with lower total brain and frontal gray matter and higher WMH volumes compared to those with low CVD risk. High CVD risk was additionally associated with lower total cortical gray matter and parietal volumes and larger lateral ventricle volumes. Men tended to have greater CVDRF-related differences in total brain volumes than women. The association of CVD risk factors on total brain volumes increased with age, equal to an approximate 7-year increase in total brain aging among the high-CVD-risk group compared to the low-risk group. The presence of infarct(s) was associated with lower total brain volumes, which was equal to an approximate 5-year increase in brain aging compared to individuals without infarcts. Infarcts were also associated with smaller total cortical gray matter, frontal and parietal volumes, and larger lateral ventricle and WMH volumes.

**Conclusion:**

The high prevalence of CVD risk among Hispanic/Latino adults may be associated with accelerated brain aging.

## Introduction

Cardiovascular disease (CVD) risk factors (e.g., hypertension) are common, and their prevalence increases with age (Tsao et al., 2022). Evidence of cerebrovascular brain injury, as clinically manifested by stroke or magnetic resonance imaging (MRI) measures such as white matter hyperintensities and clinically silent infarction, is also more prevalent with increasing age (DeCarli et al., 2005; Lamar et al., 2022). Even in the absence of stroke, CVD risk factors accelerate brain aging (Seshadri et al., 2004) through a variety of proposed mechanisms, including, but not limited to, increased inflammation, microstructural damage, exposure to toxins through the breakdown of the blood–brain barrier, and reduced cerebral blood flow (Zlokovic, 2005; Gorelick et al., 2011; Verstynen et al., 2013). As a result, individual and aggregate measures of CVD risk factors have been associated with smaller brain volumes, larger ventricular volumes, and increased risk for future cognitive impairment, stroke, dementia, and death (Jeerakathil et al., 2004; Seshadri et al., 2004; Brickman et al., 2008; Debette et al., 2010, 2011, 2019). Furthermore, certain brain regions (e.g., the frontal lobes and hippocampus) may be particularly susceptible to damage from CVD risk factors (Raz et al., 2007; Srinivasa et al., 2016). Notably, the relationship between CVD risk factors and brain injury is not uniform and may differ by sex, with women (in human and animal models) tending to experience worse brain and cognitive outcomes in the presence of CVD risk factors compared to men (Salinero et al., 2020; Huo et al., 2022).

Much of what we know about CVD risk factors and the brain in the United States, however, is from studies of non-Hispanic white (DeCarli et al., 2005) and/or Black African American cohorts (Aggarwal et al., 2010; Gottesman et al., 2015). Hispanic/Latino middle-aged and older adults living in the United States have a prevalence of CVD risk factors, which is often equal to or higher than rates found among their non-Hispanic/Latino white counterparts (Daviglus et al., 2014; Rodriguez et al., 2014). Additionally, Hispanic/Latino adults may be more susceptible to CVDRF-related differences in cognition, brain structure, and pathology compared to their non-Hispanic/Latino white peers (Filshtein et al., 2019; Stickel, 2019; Stickel et al., 2019). In fact, recent work in the Study of Latinos—Investigation of Neurocognitive Aging (SOL-INCA) finds that CVD risk factors are strongly associated with cognitive performance and MCI diagnosis (Gonzalez et al., 2019, 2020; Lamar et al., 2019; Tarraf et al., 2020). Conversely, Hispanic/Latino adults tend to have a lower prevalence of infarct compared to their non-Hispanic/Latino Black peers but similar rates as non-Hispanic/Latino whites (Prabhakaran et al., 2008) though incidence rates of infarct and stroke may be higher among Hispanic/Latino individuals (Morgenstern et al., 1997; Uchino et al., 2004). Finally, differences in stroke mortality between Hispanic/Latino adults and other ethnic and racial groups are mixed (Sacco et al., 1991; Morgenstern et al., 1997; Hartmann et al., 2001). Hispanic/Latino adults tend to have strokes at earlier ages compared to non-Hispanic/Latino white adults and may have higher prevalence, but this varies widely by age, sex, type of stroke, and heritage group (e.g., Mexican vs. Cuban heritage) (Morgenstern et al., 1997; Uchino et al., 2004; Prabhakaran et al., 2008; Rodriguez et al., 2014; Tsao et al., 2022). Heritage group alone is associated with differences in CVD risk factors, self-reported stroke, and brain structure (Daviglus et al., 2012; Stickel et al., 2023), but most existing studies of Hispanic/Latino brain aging sample almost exclusively from one or two heritage groups.

Key characteristics distinguish Hispanic/Latino stroke survivors from their non-Hispanic/Latino peers, such as lower access to healthcare and higher prevalence of certain CVD risk factors (Smith et al., 2003; Mahajan et al., 2021). Consequently, CVD risk factors are more likely to go undiagnosed and untreated for longer periods of time among Hispanic/Latino individuals, increasing the risk for more serious cardiovascular health problems and stroke (Rodriguez et al., 2014). Taken together, this highlights the critical need to further investigate the relationships between CVD risk factors, brain infarcts (a risk factor for future stroke independent of CVD risk factors) (Debette et al., 2010), and brain outcomes within diverse samples of Hispanic/Latino adults.

We, therefore, examined whether CVD risk factors and brain infarcts detected on MRI were each associated with brain volumes, lateral ventricle volumes, and white matter hyperintensity volumes (WMHs) while accounting for heritage. We also investigated if these relationships differed by age or sex. Consistent with previous findings in non-Hispanic/Latino white (DeCarli et al., 2005) and mixed racial and ethnic cohorts (Aggarwal et al., 2010), we hypothesized that higher CVD risk and the presence of infarcts would be associated with smaller brain (global and regional) volumes and larger lateral ventricle and WMH volumes. We also predicted that these exposures would exacerbate age-related brain differences as previously described in the non-Hispanic/Latino white population and would be more pronounced among women compared to men.

## Methods

### Data

The Hispanic Community Health Study/Study of Latinos (HCHS/SOL) is a population-based prospective cohort study of community-dwelling diverse Hispanic/Latino adults. During Visit 1 (2008–2011), 16,415 Hispanic/Latino adults (18–74 years) were enrolled from four major metropolitan cities: Bronx, NY; Chicago, IL; Miami, FL; and San Diego, CA. Regions that were sampled were defined by tracts from the 2000 census to ensure diversity across Hispanic/Latino heritage groups (LaVange et al., 2010). HCHS-SOL designed complex survey sampling weights that allow for generalization to the target Hispanic/Latino population. Study methods and the design are published elsewhere (Sorlie et al., 2010). The Study of Latinos-Investigation of Neurocognitive Aging Magnetic Resonance Imaging (SOL-INCA-MRI) is an ancillary study of HCHS/SOL that investigates brain health in the Hispanic/Latino community. SOL-INCA-MRI participant selection was enriched for individuals with cognitive impairment based on the National Institute on Aging Alzheimer’s Association Criteria (McKhann et al., 2011; Gonzalez et al., 2019), and the cognitively healthy subjects were randomly sampled with sex and field center matching to the participants with cognitive impairment. In addition, younger (35 to 50 years old at Visit 2) individuals were chosen at random from the parent HCHS/SOL study to obtain a lifespan perspective on Hispanic/Latino brain health. We used all available data up to 10 April 2022, excluding individuals younger than 50 years. The study included an unweighted total of 1,886 participants (weighted as 42% men and 58% women), aged 50–85 years, all of whom had completed MRI imaging and relevant image processing. Neuroimaging methods are described below and in our first report on age and sex associations with brain volumes (Stickel et al., 2023).

### MRI acquisition and analysis

Brain images were obtained on 3 T MRI scanners [GE 3 T 750 (3 sites) or Philips 3 T Achieva TX (1 site)]. The current study was interested in high-resolution T1-weighted structural images (1 mm^3^) and fluid-attenuated inversion recovery sequences (3DFLAIR). All images were processed and analyzed using pipelines created at UC Davis’ Imaging of Dementia and Aging (IDeA) laboratory. These steps are detailed elsewhere (Stickel et al., 2023). The presence of MRI infarction was determined from the size, location, and imaging characteristics of the lesion as previously described (DeCarli et al., 2005). The image analysis system allowed for superimposition of the subtraction image, the proton density image, and the T2 weighted image at a three-times magnified view, aiding in the interpretation of lesion characteristics. The signal void, best seen on T2-weighted images, was interpreted to indicate a vessel. Only lesions 3 mm or larger qualified for consideration as cerebral infarcts. Other necessary imaging characteristics included the following: (1) CSF signal characteristics on the subtraction image and (2) if the infarct was in the basal ganglia area, distinct separation from the circle of Willis vessels. Kappa values for agreement among raters were generally good and ranged from 0.73 to 0.90.

### Outcomes

The outcomes included total brain, total cortical gray matter, individual cortical lobar (frontal, parietal, temporal, and occipital), hippocampal, lateral ventricle, and total WMH volumes. All MRI measures were residualized to total cranial volume and standardized (z-scored) to facilitate interpretation across outcomes using the full SOL-INCA-MRI sample, including individuals 30–50 years of age. Lateral ventricle and total WMH measures were naturally log-transformed before residualization to normalize variance.

### Primary exposures

The Framingham cardiovascular risk score (FRS) was assessed using the Framingham 10-year risk score equation, which accounts for a wide array of CVD risk factors such as diabetes and cholesterol and is more accurate to current cardiovascular health than relying solely on self-reported measures (D’agostino et al., 2008). The FRS score is generated using male- and female-specific equations. Three CVD risk categories were generated based on FRS risk (<10% = low, 10– < 20% = medium, and ≥ 20% = high). For this study, we used CVD risk at baseline, an average of 10.1 ± 1.4 years before MRI. Evidence of infarct(s) on MRI (0 = absent, 1 = present) was operationalized as a binary measure and determined as described above.

### Covariables

The covariables included age at MRI visit, sex (male and female), and Hispanic/Latino heritage (Central American, Cuban, Dominican, Mexican, Puerto Rican, and South American).

### Analytic sample

As of April 2022, a total of 2,288 participants had MRI data collected and processed. Sampling weights were generated for this SOL-INCA MRI sample to improve generalizability to the target population. We excluded 249 participants aged less than 50 years, 3 participants with missing Hispanic/Latino heritage, 11 participants with missing Framingham score, and 139 participants with missing MRI infarcts data. Participants less than 50 years old were excluded due to limited variability in FRS and infarct, which would have resulted in over-extrapolation of the data. The final unweighted analytic sample is 1,886.

### Analytic strategy

All estimates were computed using Stata 17 software, and analyses were performed using complex survey design features to account for the SOL-INCA-MRI study design and allow appropriate generalization of the target population. First, we generated descriptive statistics by sex groupings across our covariables of interest and exposures of interest (Table 1). Table 1 includes prevalence and standard errors for categorical measures and means and standard deviations for continuous measures. To test differences by sex, we used survey-adjusted chi-squared and F-tests for categorical and continuous variables, respectively. Second, we fit survey-weighted linear regression models to test the associations between our exposures of interest and brain measure outcomes adjusting for sex, age, and Hispanic/Latino heritage. Betas and 95% confidence intervals from these models are presented in Tables 2, 3. We re-estimated all models to include two-way interactions between CVD risk and infarcts with sex and age to test for modifications in associations between cardiovascular risk and infarcts prevalence by men and women and over the SOL-INCA-MRI age continuum. ANOVA-like F-tests were used to determine the significance of interactions and are presented in Supplementary Table S1. To aid in the interpretation of results, we plotted post-hoc marginal estimates and 95% confidence intervals for main effects and significant modification effects from two- and three-way interactions with sex and age. In sensitivity models, we refit our linear models using three-way interactions between CVD risk and infarcts, independently, and age and sex (e.g., CVD risk*sex*age). The ANOVA-like F-tests derived from these models are included in Supplementary Tables S3, S5.

**Table 1:** Descriptive statistics for Study of Latinos—Investigation of Neurocognitive Aging MRI target population.

	Female	Male	Total	*p*-Value
Unweighted *N*	1,304	582	1,886	
Weighted %	58.3	41.7	100	

Age				
50–59	20.99 (1.35)	19.99 (2.22)	20.58 (1.19)	*p* = 0.741
60–69	36.09 (1.81)	38.78 (3.49)	37.21 (1.72)	
70+	42.92 (2.28)	41.22 (4.60)	42.21 (2.27)	
Framingham cardiovascular risk score				
Low	55.15 (2.36)	18.65 (2.14)	39.92 (1.93)	*p* < 0.001
Medium	26.35 (2.15)	38.35 (3.30)	31.36 (1.82)	
High	18.50 (2.33)	43.00 (4.10)	28.72 (2.42)	
Infarct(s)				
No	92.93 (1.07)	87.24 (2.40)	90.55 (1.20)	*p* = 0.012
Yes	7.07 (1.07)	12.76 (2.40)	9.45 (1.20)	
Hispanic/Latino Heritage				
Dominican	10.59 (1.22)	5.20 (1.21)	8.34 (0.97)	*p* = 0.033
Central American	9.38 (1.25)	7.35 (1.52)	8.53 (0.97)	
Cuban	25.62 (2.80)	34.39 (4.37)	29.28 (2.72)	
Mexican	25.27 (2.16)	26.97 (3.11)	25.98 (2.01)	
Puerto-Rican	17.82 (1.72)	17.09 (2.53)	17.52 (1.50)	
South American	8.40 (1.44)	5.07 (0.99)	7.01 (0.95)	
Mixed/Other	2.92 (0.70)	3.93 (1.47)	3.34 (0.74)	

Age (years)	67.72 ± 9.12	67.40 ± 6.92	67.59 ± 8.24	*p* = 0.687

Infarct(s) assessed on magnetic resonance imaging scan.

MRI, magnetic resonance imaging; SE, standard error.

**Table 2:** Associations of baseline Framingham cardiovascular risk score with brain volumes.

	Total brain volume	Hippocampus	Log lateral ventricle	Log WMH
	b/ci95	b/ci95	b/ci95	b/ci95
FRS				
Low	ref	ref	ref	ref
Medium	−0.16* [−0.29;−0.02]	0.08 [−0.10;0.26]	0.13 [−0.03;0.29]	0.22*** [0.09;0.35]
High	−0.22* [−0.39;−0.05]	0.06 [−0.18;0.30]	0.30** [0.11;0.49]	0.41*** [0.21;0.61]

	Combined gray	Frontal cortical gray	Occipital cortical gray	Temporal cortical gray	Parietal cortical gray
	b/ci95	b/ci95	b/ci95	b/ci95	b/ci95
FRS					
Low	ref	ref	ref	ref	ref
Medium	−0.13 [−0.27;0.02]	−0.18* [−0.31;−0.04]	−0.01 [−0.18;0.16]	−0.01 [−0.18;0.15]	−0.10 [−0.27;0.06]
High	−0.34*** [−0.53;−0.15]	−0.42*** [−0.61;−0.23]	−0.19 [−0.45;0.06]	0.02 [−0.15;0.19]	−0.29* [−0.52;−0.05]

Models adjusted for age, sex, and Hispanic/Latino heritage group.

b, beta; ci, confidence interval; FRS, Framingham Risk Score; MRI, magnetic resonance imaging; Std = standardized residualized for cranial size; WMH, white matter hyperintensity.

*p < 0.05; **p < 0.01; ***p < 0.001.

**Table 3:** Associations of infarcts with brain volumes.

	Total brain volume	Hippocampus	Log lateral ventricle	Log WMH
	b/ci95	b/ci95	b/ci95	b/ci95
Infarcts				
No	ref	ref	ref	ref
Yes	−0.35** [−0.57;−0.12]	0.16 [−0.09;0.41]	0.40** [0.14;0.66]	0.63*** [0.41;0.85]

	Combined gray	Frontal cortical gray	Occipital cortical gray	Temporal cortical gray	Parietal cortical gray
	b/ci95	b/ci95	b/ci95	b/ci95	b/ci95
Infarcts					
No	ref	ref	ref	ref	ref
Yes	−0.35** [−0.57;−0.13]	−0.31** [−0.54;−0.08]	−0.27 [−0.57;0.04]	−0.15 [−0.32;0.03]	−0.27* [−0.48;−0.05]

Infarct(s) assessed on magnetic resonance imaging scan.

Models adjusted for age, sex, and Hispanic/Latino heritage group.

b, beta; ci, confidence interval; MRI, magnetic resonance imaging; Std, standardized residualized for cranial size; WMH, white matter hyperintensity.

*p < 0.05; **p < 0.01; ***p < 0.001.

## Results

### Descriptives

A total of 582 men and 1,304 women (unweighted) were included in the analysis. Women had lower baseline cardiovascular risk as assessed by the Framingham cardiovascular risk score and lower prevalent rates of infarcts compared to men (Table 1).

### Framingham cardiovascular risk score

**Figure 1:**
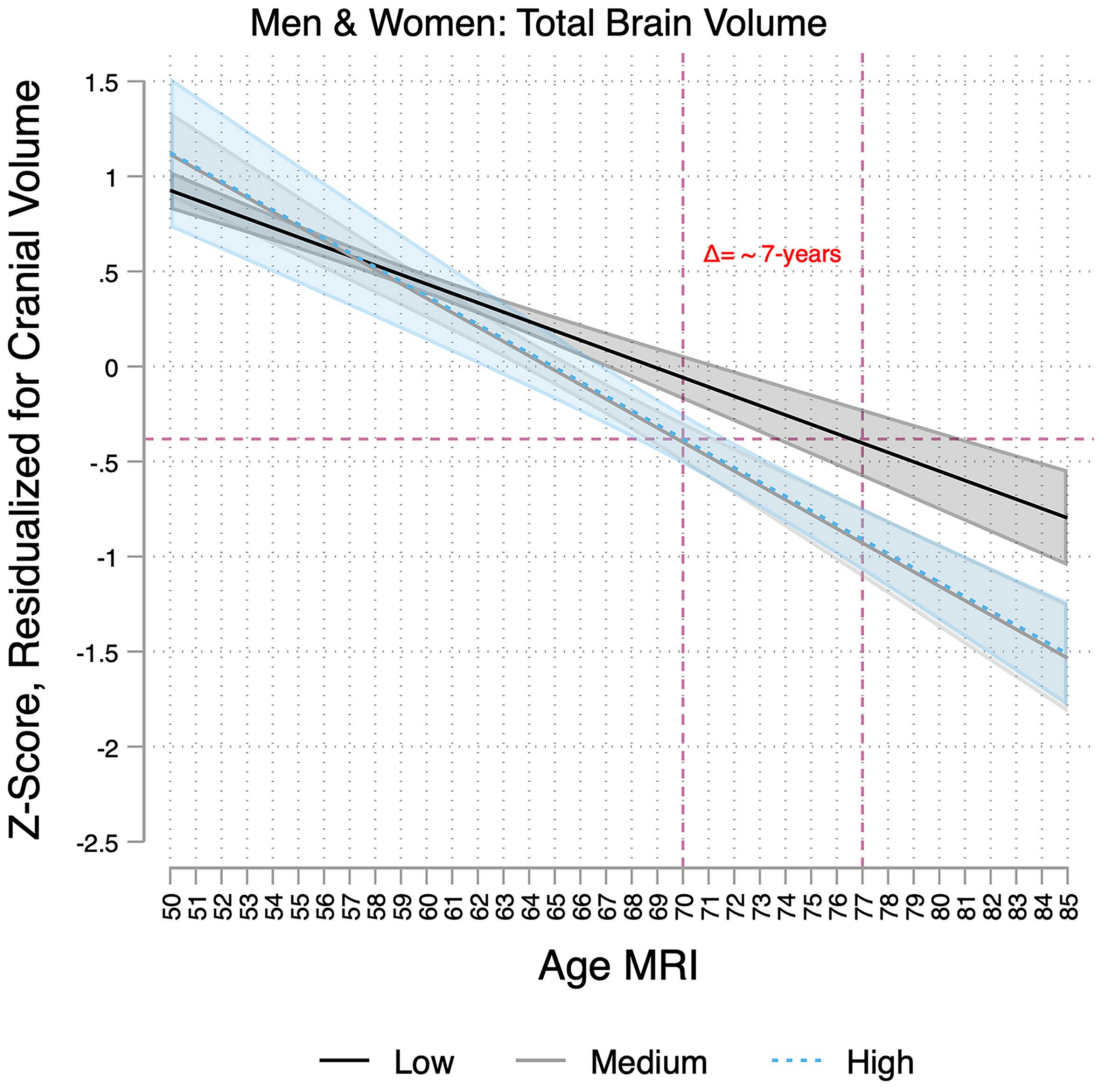
Associations between age and brain volumes per baseline Framingham cardiovascular risk score group (low, medium, and high) in the Study of Latinos-Investigation of Neurocognitive Aging Magnetic Resonance Imaging (SOL-INCA MRI). Estimates represent the associations of age with total brain volumes per cardiovascular disease risk group (low, medium, and high). These are derived from models that adjusted for sex and Hispanic/Latino heritage groups and include an age × cardiovascular risk interaction. The dotted lines demonstrate that a 70-year-old with high cardiovascular risk has similar total brain volumes to that of a 77-year-old at low risk, suggesting 7 years of brain aging in the former group.

As expected (Tsao et al., 2023), CVD risk, obtained on average 10.1 years before MRI, increased with age (β_age_ = −0.007; 95%CI [0.002;0.006]) and male sex (β_men_ = 0.04; 95%CI [0.038;0.045]). Medium CVD risk based on the FRS was associated with smaller total brain matter (β_total_brain_ = −0.16; 95%CI [−0.29;−0.02]). Age-adjusted differences were also significantly smaller for frontal gray matter and larger for WMH volumes than low CVD risk. High CVD risk was additionally associated with larger lateral ventricles, smaller total gray, and smaller parietal gray matter volumes compared to low CVD risk (Table 2; Figure 1). No significant associations were found between the levels of CVD risk with hippocampal, occipital, or temporal volumes (Table 2).

In two-way interaction models, we detected a significant age by sex and CVD risk by sex interaction but only for total brain volumes (Supplementary Table S1). Individuals with medium and high CVD risk had more pronounced decrements in total brain volumes with older age (Supplementary Table S2). For example, this difference translates into a 7-year increase in brain aging for some individuals (e.g., a 70-year-old in the highest CVD risk group had a predicted brain volume equivalent to a 77-year-old in the lowest risk group; Figure 1). Men with medium and high CVD risk had significantly lower total brain volume (Supplementary Table S2). We did not find consistent three-way interactions (CVD risk*Sex*Age) on brain markers (Supplementary Tables S3, S4).

### MRI infarcts

**Figure 2:**
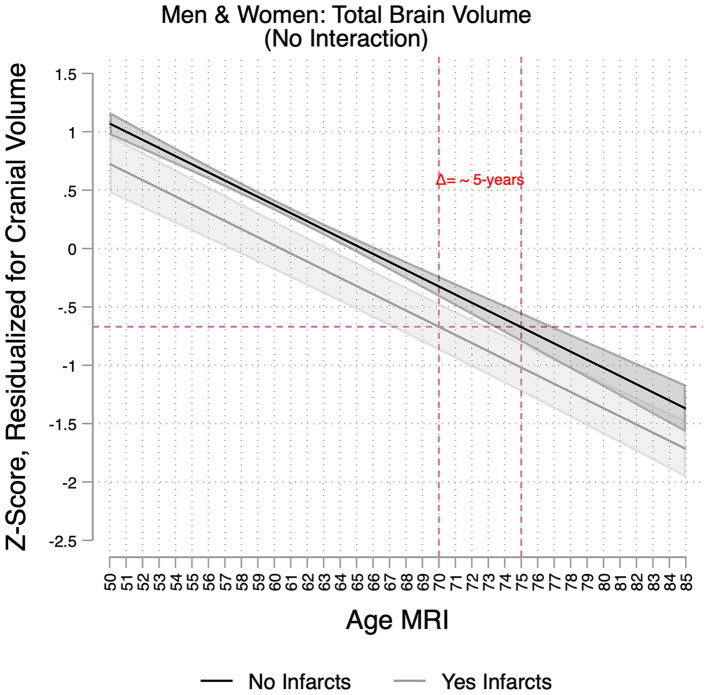
Association between age and total brain volumes per infarct group in the Study of Latinos-Investigation of Neurocognitive Aging Magnetic Resonance Imaging (SOL-INCA MRI). Infarct(s) assessed on magnetic resonance imaging scan. Estimates represent the main associations between age and total brain volumes per infarct group, and these are derived from models that included main effects for sex and Hispanic/Latino heritage groups. The dotted lines demonstrate that a 70-year-old with the presence of infarct(s) has similar total brain volumes to that of a 75-year-old without infarcts, suggesting 5 years of brain aging in the former group.

**Figure 3:**
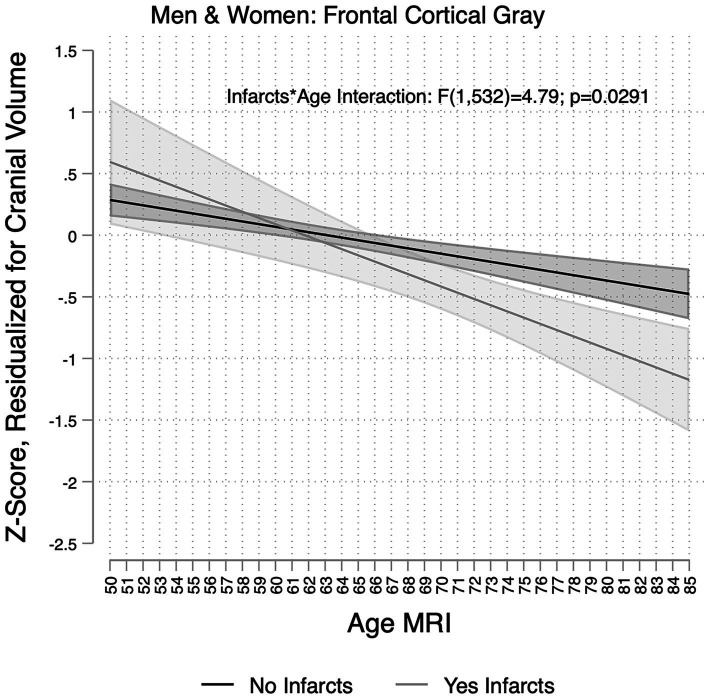
Association between age and frontal gray matter volumes per infarct group in the Study of Latinos-Investigation of Neurocognitive Aging Magnetic Resonance Imaging (SOL-INCA MRI). Infarct(s) assessed on magnetic resonance imaging scan. Estimates represent the main associations between age and frontal gray matter volumes per infarct group, and these are derived from models that included main effects for sex and Hispanic/Latino heritage groups and an age × infarct interaction.

MRI infarcts were also associated with lower residual total brain volume (β_Δtotal_brain_ = −0.35; 95%CI [−0.57;−0.12]). For someone 70 years of age with an infarct, this translates to approximately a 5-year increase in brain aging (Figure 2). Smaller total gray matter and frontal and parietal volumes, as well as larger lateral ventricle and WMH volumes, were also significantly associated with MRI infarction (Table 3). Sex did not modify the associations between infracts and brain measures. MRI infarcts, however, were linked to more pronounced decrements in frontal gray volumes with older age (Figure 3, Supplementary Tables S1, S2). Three-way interaction sensitivity models indicated that age decrements in frontal cortical gray volumes were specifically evident for men with MRI infarcts (Supplementary Tables S4, S5, Supplementary Figure S1). No other interactions were significant.

### Relationships between heritage and brain volumes

The impact of heritage differed significantly by brain region after adjusting for age, sex, and CVD or infarcts (Supplementary Figures S2–S6). Individuals of Dominican and Puerto Rican heritage had larger WMH volumes compared to all other groups except the mixed/other heritage group (Supplementary Figure S2). Dominicans had larger cortical gray matter and frontal and occipital volumes compared to all other heritage groups (Supplementary Figures S3–S5). Puerto Ricans had larger cortical and frontal gray matter volumes than those of Central American, Cuban, Mexican, and South American heritage. They also had larger occipital gray matter volumes compared to Cubans and Mexicans. Dominicans had larger parietal volumes compared to all other groups except those of mixed/other heritage, whereas Puerto Ricans had larger volumes in this region compared to Cubans and Mexicans (Supplementary Figure S6).

## Discussion

Our cross-sectional analysis of over 1,800 Hispanic/Latino individuals 50 years of age and older indicates that medium and high CVD risk, determined on average 10 years before MRI and occurring in nearly 40% of the cohort, was associated with smaller total brain and cortical gray matter volumes, larger lateral ventricles, and larger WMH volumes. High CVD risk was translated to approximately 7 years of total brain aging compared to low CVD risk. Additional findings include the following: (1) associations between CVD risk and total brain volumes were more prominent among men than women; (2) persons identified as Dominican or Puerto Rican tended to have significantly larger regional brain volumes for the degree of CVD risk; and (3) the associations of CVD risk and MRI infarction each showed regional specificity. Given that vascular risk can be medically managed (Lloyd-Jones et al., 2010, 2022) and improved management is associated with reduced heart disease and stroke (Lewis et al., 2021; Tsao et al., 2023) as well as reduced accumulation of WMH (The SPRINT MIND Investigators for the SPRINT Research Group, 2019) and incidence of cognitive impairment (Williamson et al., 2019),CVD risk, management, and prevention in the Hispanic/Latino population (Smith et al., 2003) could, in turn, prevent brain injury and have widespread benefit on brain aging, including future stroke and dementia, in this underserved population.

### Impact of CVD risk on MRI measures

Consistent with existing literature (Jeerakathil et al., 2004; Seshadri et al., 2004; Brickman et al., 2008; Srinivasa et al., 2016), our results indicated that both medium and high CVD risk groups were associated with smaller total brain, gray matter, and frontal brain volumes as well as larger WMH volumes compared to the low CVD risk group. In the predominantly non-Hispanic/Latino white Framingham Offspring Study, higher CVD risk was associated with smaller total brain (Seshadri et al., 2004), larger WMH volumes, and lower cognitive performance (Elias et al., 2004; Jeerakathil et al., 2004; Au et al., 2006). Using an aggregate count of cardiovascular disease factors in a cohort of African American, Caribbean Hispanic/Latino, and non-Hispanic/Latino white adults, Brickman and colleagues detected similar relationships, which did not vary by race or ethnicity (Brickman et al., 2008). Previous literature has suggested that the frontal lobes are particularly sensitive to a wide range of CVD risk factors (Raz et al., 2003; Birdsill et al., 2013), further confirming the consistency of our findings. Existing literature has detected step-wise increases in risk for WMHs when comparing healthy, well-controlled, and uncontrolled CVD risk (de Leeuw et al., 2001). In contrast, only the high CVD risk group had smaller parietal volumes and larger lateral ventricular volumes compared to the low-risk group, suggesting that these brain regions may be more resilient to medium levels of CVD risk factors. In a mixed racial and ethnic sample, higher self-reported CVD risk was associated with smaller total brain and higher WMH volumes but was not linked to lateral ventricle volumes, supporting that the latter is more resilient to CVD risk factors and precise biometrics may be needed to detect CVD risk-based differences (Brickman et al., 2008).

CVD risk factors may exacerbate brain aging among Hispanic/Latino adults. Consistent with prior studies showing that midlife CVD risk factors impact brain structure and dementia risk in later life (Debette et al., 2010, 2011; Meng et al., 2014), medium and high CVD risks were associated with more pronounced age-related differences (lower volumes at older ages) in total brain volumes than the low CVD risk group. Notably, this potential increased negative impact of CVD risk factors at older ages was not found for regional brain or WMH volumes, suggesting that this is a cumulative effect. Several other factors may contribute to variance at the regional level (e.g., age of onset and duration of CVD risk factors), but testing such hypotheses is beyond the scope of this manuscript.

Similarly, sex also appeared to have a global impact on the relationships between CVD risk factors and brain volumes. Specifically, men appeared to have smaller brain volumes at medium and high levels of CVD risk factors compared to low CVD risk, whereas women demonstrated more stability in total brain volumes across CVD risk groups. It is possible that certain factors (e.g., estrogen exposure) may have more influence on brain volumes in women and may be contributing to this higher variability compared to men (Li et al., 2014). Overall, sex differences were subtle, and it is important to note that women had larger confidence intervals than men, particularly at high CVD risk. Surprisingly, sex did not modify susceptibility to WMHs in the presence of CVD risk, despite our previous work (Stickel et al., 2023) and others (Sachdev et al., 2009) showing that women may be particularly vulnerable to higher WMH volumes with advancing age. Additionally, recent study suggests that even before older adulthood, cardiovascular disease risk is associated with cognitive declines among women more so than men (Huo et al., 2022). Importantly, the FRS score captures sex (D’agostino et al., 2008), so our findings may be conservative relative to other studies that include CVD risk measures that do not already account for sex. This may also explain why sex differences in the relationships between FRS and WMH volumes were not detected in the Framingham Offspring Study (Jeerakathil et al., 2004).

### Impact of MRI infarcts on MRI measures

The overall prevalence rate of infarcts in the present study (9%) is somewhat lower than in other studies (Fanning et al., 2014), although there was a significant age-specific increase in prevalence, reaching approximately 15% among the oldest members of the cohort. Consistent with previous studies, men had a higher age-adjusted prevalence (DeCarli et al., 2005). Differences between our study and other reports, therefore, may be explained by the methods and generally younger age of our cohort (mean age = 68 years). For example, although the average age in the Atherosclerosis Risk in Communities (ARIC) study was 62 years, the reported 15% prevalence of infarcts included infarcts less than 3 mm, whereas our study did not (Gottesman et al., 2015). In the Northern Manhattan Study, infarct prevalence was 24, 18, and 16% for African American, non-Hispanic/Latino white, and Caribbean Hispanic/Latino adults, respectively, using the same sizing criteria as the present study but with a nominally older sample (mean age = 71 years) (Prabhakaran et al., 2008). Notably, infarct prevalence was lower among younger participants (7% for Caribbean Hispanic/Latino individuals under 65 years) and those born outside of the United States (14%) (Prabhakaran et al., 2008). Consistent with the Framingham Heart Study (DeCarli et al., 2005), we found that the presence of infarct(s) was associated with smaller total brain, gray matter, frontal and parietal lobe volumes, and larger lateral ventricle and WMH volumes after accounting for relevant demographic variables, including age. The presence of MRI infarction was also associated with greater age-related differences in MRI volumes, particularly in the frontal cortex. In the Framingham Heart Study (DeCarli et al., 2005), the presence of infarct(s) was associated with more prominent age-related differences in total brain, temporal lobe, and lateral ventricle volumes. In the present study, we may detect an exacerbation of the normal aging process, as the frontal lobes are susceptible to age-related volume loss (Fjell et al., 2009; Pfefferbaum et al., 2013). Although we did not detect any modifications by sex alone, evidence of exacerbated aging in the frontal cortex appeared to be driven by the men in our sample. Similarly, in the Framingham Heart Study, only men (not women) had smaller total brain volumes in the presence of infarct(s) (DeCarli et al., 2005). Additionally, individuals identified as having infarct(s) were more likely to be men and older. Thus, we may have been underpowered to detect such modifications. As the cohort continues to age and infarct incidents occur, we may have more power to detect associations. Alternatively, the lack of sex modification may be a result of the complex factors that impact infarction by sex (Volgman et al., 2019), including but not limited to (1) greater longevity among women, which increases the risk of infarction, (2) mixed evidence of a protective effect of estrogen exposure to reduce risk of infarction (Alonso de Lecinana et al., 2007) and potentially improve recovery post-infarction (Roof and Hall, 2000) among women compared to men, and (3) more drastic fluctuations in estrogen among women than men surrounding menopause (Zarate et al., 2017).

### Study limitations

These findings should be interpreted in the context of the limitations of this study. First, these data are cross-sectional and, therefore, represent differences between people of various ages. Future studies will examine longitudinal data to better characterize brain changes with age among Hispanic/Latino individuals. Second, we do not have information on the duration of CVD risk factors or age at infarct, which limits our inference related to age-specific effects on brain health (Pase et al., 2018). Third, the age range among low CVD risk men was relatively truncated. This likely reflects the small proportion of low CVD risk, older Hispanic/Latino men in the U.S. (Daviglus et al., 2016). Fourth, we did not have enough participants with more than one infarct to examine the relationships between infarct count and brain outcomes. Despite these limitations, our study has several strengths, including (1) a large sample size selected to be representative of the Hispanic/Latino population in four major metropolitan areas, (2) self-identification of heritage, (3) a validated measure of CVD risk factors, and (4) MRI-measurements of the infarct.

## Conclusion

Using data from over 1,800 Hispanic/Latino adults enrolled in the SOL-INCA-MRI, we found smaller brain and larger lateral ventricle and WMH volumes associated with medium and high levels of CVD risk and the presence of infarct(s) on MRI. Some of these relationships varied by age, sex, and neuroanatomical location. That is, medium and high CVD risk factors were each associated with exacerbated age-related differences in total brain volumes, and men tended to have greater CVDRF-related differences in total brain volumes than women. Additionally, the presence of infarct(s) was associated with larger age-related differences in frontal cortical volumes, particularly among men. Given the high prevalence of moderate and high CVD risk and the previously described relationship between CVD risk factors and cognition (Gonzalez et al., 2019; Lamar et al., 2019; Tarraf et al., 2020) in this population, intervention strategies to reduce the impact of CVD risk on brain injury and cognition (The SPRINT MIND Investigators for the SPRINT Research Group, 2019; Williamson et al., 2019) may have considerable benefit for Hispanic/Latino adults living in the United States.

## Data availability statement

The datasets presented in this article are not readily available because Data from the Hispanic Community Health Study/Study of Latinos (HCHS/SOL) and SOL-Investigation of Neurocognitive Aging (SOL-INCA) are available at https://biolincc.nhlbi.nih.gov/studies/hchssol/. Magnetic resonance imaging data is not currently available. Requests to access the datasets should be directed to https://biolincc.nhlbi.nih.gov/studies/hchssol/.

## Ethics statement

The studies involving humans were approved by Institutional Review Boards at each of the participating sites in the Bronx (Albert Einstein), Chicago (University of Illinois), Miami (University of Miami), and San Diego (San Diego State University) approved this project. The studies were conducted in accordance with the local legislation and institutional requirements. The participants provided their written informed consent to participate in this study.

## Author contributions

AS: Conceptualization, Writing – original draft, Writing – review & editing. WT: Conceptualization, Data curation, Formal analysis, Methodology, Supervision, Validation, Visualization, Writing – original draft, Writing – review & editing. KG: Writing – review & editing. AP: Writing – review & editing. DZ: Writing – review & editing. JC: Writing – review & editing. CI: Writing – review & editing. RK: Writing – review & editing. RL: Validation, Writing – review & editing. MD: Writing – review & editing. FT: Writing – review & editing. ML: Writing – review & editing. LG: Writing – review & editing. GT: Writing – review & editing. MG: Writing – review & editing. AR: Writing – review & editing. VI: Data curation, Methodology, Writing – review & editing. SS: Data curation, Methodology, Writing – review & editing. HG: Conceptualization, Funding acquisition, Project administration, Resources, Supervision, Writing – review & editing. CD: Conceptualization, Data curation, Funding acquisition, Methodology, Project administration, Resources, Software, Supervision, Writing – original draft, Writing – review & editing.
