# Supplementary material for: Cardiovascular disease risk exacerbates brain aging among Hispanic/Latino adults in the SOL-INCA-MRI Study
Source: Front Aging Neurosci. 2024 May 8;16:1390200. doi: 10.3389/fnagi.2024.1390200 (PMC11110680; doi:10.3389/fnagi.2024.1390200)
Supplement: Supplementary file 1 [file Data_Sheet_1.docx]

Supplemental Table 1. Wald (F)-tests for main effects and sex and age interactions with baseline Framingham Cardiovascular Risk Scores and infarcts, independently.

|  | **FRS** | | | | **Infarcts** | | |
| --- | --- | --- | --- | --- | --- | --- | --- |
| **Outcome** | **Exposure** | **Main Effects Only**  **F;p-value;(degrees of freedom)** | **Interactions**  **F;p-value;(degrees of freedom)** | **Exposure** | | **Main Effects Only**  **F;p-value;(degrees of freedom)** | **Interactions***  **F;p-value;(degrees of freedom)** |
| **Total Brain Volume** | |  |  |  | |  |  |
|  | FRS | F=4.15;p=0.016;(2;531) |  | Infarcts | | F=9.18;p=0.003;(1;532) |  |
|  | Sex | F=11.52;p=0.001;(1;532) |  | Sex | | F=19.55;p<0.001;(1;532) |  |
|  | Age | F=208.23;p<0.001;(1;532) |  | Age | | F=279.97;p=≤0.001;(1;532) |  |
|  | FRS*Sex |  | F=5.02;p=0.007;(2;531) | Infarcts*Sex | |  | F=0.32;p=0.571;(1;532) |
|  | FRS*Age |  | F=6.12;p=0.002;(2;531) | Infarcts*Age | |  | F=0.18;p=0.675;(1;532) |
| **Hippocampus** | |  |  |  | |  |  |
|  | FRS | F=0.39;p=0.674;(2;531) |  | Infarcts | | F=1.52;p=0.218;(1;532) |  |
|  | Sex | F=0;p=0.987;(1;532) |  | Sex | | F=0.02;p=0.884;(1;532) |  |
|  | Age | F=14.63;p<0.001;(1;532) |  | Age | | F=19.91;p<0.001;(1;532) |  |
|  | FRS*Sex |  | F=0.99;p=0.371;(2;531) | Infarcts*Sex | |  | F=0.15;p=0.695;(1;532) |
|  | FRS*Age |  | F=2.89;p=0.056;(2;531) | Infarcts*Age | |  | F=0.53;p=0.467;(1;532) |
| **Log Lateral Ventricle** | |  |  |  | |  |  |
|  | FRS | F=5.22;p=0.006;(2;531) |  | Infarcts | | F=9.01;p=0.003;(1;532) |  |
|  | Sex | F=4.44;p=0.036;(1;532) |  | Sex | | F=8.18;p=0.004;(1;532) |  |
|  | Age | F=96.24;p=≤0.001;(1;532) |  | Age | | F=120.69;p=≤0.001;(1;532) |  |
|  | FRS*Sex |  | F=1.15;p=0.318;(2;531) | Infarcts*Sex | |  | F=2.24;p=0.135;(1;532) |
|  | FRS*Age |  | F=1.43;p=0.241;(2;531) | Infarcts*Age | |  | F=1.49;p=0.222;(1;532) |
| **Log WMH** | |  |  |  | |  |  |
|  | FRS | F=9.62;p<0.001;(2;531) |  | Infarcts | | F=31.27;p<0.001;(1;532) |  |
|  | Sex | F=1.72;p=0.191;(1;532) |  | Sex | | F=0;p=0.978;(1;532) |  |
|  | Age | F=93.89;p<0.001;(1;532) |  | Age | | F=168.06;p<0.001;(1;532) |  |
|  | FRS*Sex |  | F=2.31;p=0.1;(2;531) | Infarcts*Sex | |  | F=1.96;p=0.162;(1;532) |
|  | FRS*Age |  | F=0.58;p=0.559;(2;531) | Infarcts*Age | |  | F=1.07;p=0.301;(1;532) |
| **Combined Gray** | |  |  |  | |  |  |
|  | FRS | F=6.12;p=0.002;(2;531) |  | Infarcts | | F=9.4;p=0.002;(1;532) |  |
|  | Sex | F=1.72;p=0.19;(1;532) |  | Sex | | F=6.46;p=0.011;(1;532) |  |
|  | Age | F=44.22;p<0.001;(1;532) |  | Age | | F=79.87;p<0.001;(1;532) |  |
|  | FRS*Sex |  | F=1.57;p=0.209;(2;531) | Infarcts*Sex | |  | F=0.42;p=0.52;(1;532) |
|  | FRS*Age |  | F=1.37;p=0.256;(2;531) | Infarcts*Age | |  | F=1.8;p=0.18;(1;532) |
| **Frontal Cortical Gray** | |  |  |  | |  |  |
|  | FRS | F=10.02;p<0.001;(2;531) |  | Infarcts | | F=6.8;p=0.009;(1;532) |  |
|  | Sex | F=2.22;p=0.137;(1;532) |  | Sex | | F=9.48;p=0.002;(1;532) |  |
|  | Age | F=9.56;p=0.002;(1;532) |  | Age | | F=32.77;p<0.001;(1;532) |  |
|  | FRS*Sex |  | F=0.11;p=0.898;(2;531) | Infarcts*Sex | |  | F=1.16;p=0.283;(1;532) |
|  | FRS*Age |  | F=2.28;p=0.104;(2;531) | Infarcts*Age | |  | **F=4.79;p=0.029;(1;532)** |
| **Occipital Cortical Gray** | |  |  |  | |  |  |
|  | FRS | F=1.33;p=0.266;(2;531) |  | Infarcts | | F=2.9;p=0.089;(1;532) |  |
|  | Sex | F=0.33;p=0.566;(1;532) |  | Sex | | F=0.07;p=0.788;(1;532) |  |
|  | Age | F=7.17;p=0.008;(1;532) |  | Age | | F=13.25;p<0.001;(1;532) |  |
|  | FRS*Sex |  | F=0.27;p=0.767;(2;531) | Infarcts*Sex | |  | F=0.22;p=0.641;(1;532) |
|  | FRS*Age |  | F=0.28;p=0.755;(2;531) | Infarcts*Age | |  | F=0.01;p=0.936;(1;532) |
| **Temporal Cortical Gray** | |  |  |  | |  |  |
|  | FRS | F=0.07;p=0.935;(2;531) |  | Infarcts | | F=2.78;p=0.096;(1;532) |  |
|  | Sex | F=0.05;p=0.816;(1;532) |  | Sex | | F=0.01;p=0.909;(1;532) |  |
|  | Age | F=73.49;p<0.001;(1;532) |  | Age | | F=77.44;p<0.001;(1;532) |  |
|  | FRS*Sex |  | F=1.11;p=0.329;(2;531) | Infarcts*Sex | |  | F=0;p=0.995;(1;532) |
|  | FRS*Age |  | F=0.56;p=0.572;(2;531) | Infarcts*Age | |  | F=1.24;p=0.266;(1;532) |
| **Parietal Cortical Gray** | |  |  |  | |  |  |
|  | FRS | F=2.92;p=0.055;(2;531) | F=0.89;p=0.413;(2;531) | Infarcts | | F=5.94;p=0.015;(1;532) |  |
|  | Sex | F=4.23;p=0.04;(1;532) | F=1.1;p=0.296;(1;532) | Sex | | F=9.57;p=0.002;(1;532) |  |
|  | Age | F=15.47;p<0.001;(1;532) | F=3.25;p=0.072;(1;532) | Age | | F=31.85;p<0.001;(1;532) |  |
|  | FRS*Sex |  | F=2.43;p=0.089;(2;531) | Infarcts*Sex | |  | F=0;p=1;(1;532) |
|  | FRS*Age |  | F=1.08;p=0.339;(2;531) | Infarcts*Age | |  | F=0.12;p=0.732;(1;532) |

Notes:

Main effects are not shown

Infarct(s) assessed on magnetic resonance imaging scan.

Models adjusted for Hispanic/Latino heritage group.

Abbreviations: FRS = Framingham Risk Score; WMH = white matter hyperintensity

Supplemental Table 2. Regression models including main effects and sex and age (2-way) interactions with baseline Framingham Cardiovascular Risk Scores and infarcts, independently.

|  | **Total Brain Volume** | **Hippocampus** | **Log Lateral Ventricle** | **Log WMH** | **Combined Gray** | **Frontal Cortical Gray** | **Occipital Cortical Gray** | **Temporal Cortical Gray** | **Parietal Cortical Gray** |
| --- | --- | --- | --- | --- | --- | --- | --- | --- | --- |
|  | b/ci95 | b/ci95 | b/ci95 | b/ci95 | b/ci95 | b/ci95 | b/ci95 | b/ci95 | b/ci95 |
| Low FRS | ref | ref | ref | ref | ref | ref | ref | ref | ref |
| Medium FRS | 1.66** [0.57;2.76] | 2.46* [0.37;4.54] | 0.13 [-1.18;1.44] | 0.94 [-0.27;2.15] | -0.61 [-1.92;0.69] | -1.33 [-2.69;0.04] | -0.65 [-2.38;1.08] | 0.63 [-0.56;1.81] | 0.10 [-1.34;1.54] |
| High FRS | 1.69* [0.27;3.12] | 1.57 [-0.87;4.00] | -1.27 [-3.07;0.53] | 0.89 [-0.63;2.42] | 0.77 [-0.72;2.26] | 0.40 [-1.09;1.89] | 0.10 [-2.12;2.32] | 0.54 [-1.52;2.60] | 1.29 [-0.65;3.23] |
| Female | ref | ref | ref | ref | ref | ref | ref | ref | ref |
| Male | 0.01 [-0.15;0.16] | 0.14 [-0.10;0.38] | 0.02 [-0.19;0.23] | 0.06 [-0.11;0.22] | -0.02 [-0.23;0.19] | -0.09 [-0.30;0.11] | 0.07 [-0.16;0.30] | 0.12 [-0.14;0.38] | -0.12 [-0.34;0.10] |
| Age | -0.05*** [-0.06;-0.04] | -0.01 [-0.02;0.01] | 0.04*** [0.03;0.05] | 0.04*** [0.03;0.06] | -0.03*** [-0.04;-0.02] | -0.02** [-0.03;-0.01] | -0.02* [-0.03;-0.00] | -0.04*** [-0.05;-0.03] | -0.01 [-0.03;0.00] |
| Low FRS*Age | ref | ref | ref | ref | ref | ref | ref | ref | ref |
| Medium FRS*Age | -0.03** [-0.04;-0.01] | -0.04* [-0.07;-0.01] | 0.00 [-0.02;0.02] | -0.01 [-0.03;0.01] | 0.01 [-0.01;0.03] | 0.02 [-0.00;0.04] | 0.01 [-0.02;0.03] | -0.01 [-0.03;0.01] | -0.01 [-0.03;0.02] |
| High FRS*Age | -0.03** [-0.05;-0.01] | -0.02 [-0.05;0.01] | 0.02 [-0.00;0.04] | -0.01 [-0.03;0.01] | -0.01 [-0.03;0.01] | -0.01 [-0.03;0.01] | -0.00 [-0.03;0.03] | -0.01 [-0.03;0.02] | -0.02 [-0.05;0.01] |
| Low FRS*Female | ref | ref | ref | ref | ref | ref | ref | ref | ref |
| Low FRS*Male | ref | ref | ref | ref | ref | ref | ref | ref | ref |
| Medium FRS*Female | ref | ref | ref | ref | ref | ref | ref | ref | ref |
| Medium FRS*Male | -0.36** [-0.61;-0.11] | -0.12 [-0.51;0.27] | 0.13 [-0.17;0.43] | -0.26* [-0.50;-0.02] | 0.04 [-0.27;0.35] | 0.02 [-0.28;0.31] | 0.07 [-0.29;0.44] | -0.09 [-0.39;0.21] | 0.15 [-0.19;0.49] |
| High FRS*Female | ref | ref | ref | ref | ref | ref | ref | ref | ref |
| High FRS*Male | -0.46* [-0.81;-0.10] | -0.35 [-0.84;0.14] | 0.34 [-0.11;0.79] | -0.17 [-0.54;0.20] | -0.27 [-0.65;0.10] | -0.07 [-0.43;0.30] | -0.12 [-0.63;0.40] | -0.34 [-0.78;0.11] | -0.35 [-0.80;0.10] |
| Intercept | 3.54*** [2.94;4.13] | 0.46 [-0.61;1.53] | -2.93*** [-3.63;-2.23] | -2.55*** [-3.26;-1.84] | 2.73*** [1.99;3.46] | 2.04*** [1.38;2.70] | 1.69*** [0.77;2.60] | 2.46*** [1.79;3.13] | 1.61*** [0.67;2.55] |

|  | **Total Brain Volume** | **Hippocampus** | **Log Lateral Ventricle** | **Log WMH** | **Combined Gray** | **Frontal Cortical Gray** | **Occipital Cortical Gray** | **Temporal Cortical Gray** | **Parietal Cortical Gray** |
| --- | --- | --- | --- | --- | --- | --- | --- | --- | --- |
|  | b/ci95 | b/ci95 | b/ci95 | b/ci95 | b/ci95 | b/ci95 | b/ci95 | b/ci95 | b/ci95 |
| No Infarcts | ref | ref | ref | ref | ref | ref | ref | ref | ref |
| Yes Infarcts | -0.70 [-2.58;1.19] | -0.52 [-2.33;1.30] | 1.91 [-0.21;4.03] | 1.69 [-0.02;3.40] | 0.95 [-0.84;2.75] | 1.83 [-0.02;3.69] | -0.08 [-2.75;2.60] | 0.72 [-0.79;2.24] | -0.63 [-2.71;1.45] |
| Female | ref | ref | ref | ref | ref | ref | ref | ref | ref |
| Male | -0.30*** [-0.45;-0.16] | 0.02 [-0.15;0.20] | 0.28** [0.11;0.45] | 0.03 [-0.10;0.15] | -0.16* [-0.31;-0.02] | -0.20** [-0.35;-0.05] | 0.04 [-0.14;0.22] | -0.01 [-0.16;0.14] | -0.24** [-0.41;-0.08] |
| Age | -0.07*** [-0.08;-0.06] | -0.03*** [-0.04;-0.01] | 0.06*** [0.05;0.07] | 0.05*** [0.04;0.06] | -0.04*** [-0.05;-0.03] | -0.02*** [-0.03;-0.01] | -0.02*** [-0.03;-0.01] | -0.04*** [-0.05;-0.03] | -0.03*** [-0.04;-0.02] |
| No Infarcts*Female | ref | ref | ref | ref | ref | ref | ref | ref | ref |
| No Infarcts*Male | ref | ref | ref | ref | ref | ref | ref | ref | ref |
| Yes Infarcts*Female | ref | ref | ref | ref | ref | ref | ref | ref | ref |
| Yes Infarcts*Male | -0.11 [-0.51;0.28] | -0.09 [-0.52;0.35] | -0.36 [-0.82;0.11] | -0.30 [-0.72;0.12] | -0.15 [-0.60;0.30] | -0.21 [-0.60;0.18] | -0.15 [-0.76;0.47] | -0.00 [-0.44;0.44] | -0.00 [-0.45;0.45] |
| No Infarcts*Age | ref | ref | ref | ref | ref | ref | ref | ref | ref |
| Yes Infarcts*Age | 0.01 [-0.02;0.03] | 0.01 [-0.02;0.04] | -0.02 [-0.05;0.01] | -0.01 [-0.04;0.01] | -0.02 [-0.04;0.01] | -0.03* [-0.05;-0.00] | -0.00 [-0.04;0.04] | -0.01 [-0.03;0.01] | 0.01 [-0.02;0.03] |
| Intercept | 4.91*** [4.31;5.51] | 1.60*** [0.80;2.40] | -3.86*** [-4.56;-3.16] | -2.85*** [-3.35;-2.36] | 3.22*** [2.59;3.85] | 2.30*** [1.69;2.92] | 1.80*** [1.01;2.60] | 2.68*** [1.99;3.36] | 2.54*** [1.81;3.26] |

Notes:

Infarct(s) assessed on magnetic resonance imaging scan.

Main effects from interaction models have limited interpretability

Models adjusted for Hispanic/Latino heritage group.

Abbreviations: b = beta; ci = confidence interval; FRS = Framingham Risk Score; WMH = white matter hyperintensity

Supplemental Table 3. Wald (F)-tests for main effects and three-way sex and age interactions with baseline Framingham Cardiovascular Risk Scores.

|  | **FRS** | | | | |
| --- | --- | --- | --- | --- | --- |
| **Brain Outcome** | **Exposure** | **F-Test** | **p-value** | **df1** | **df2** |
| ***Total Brain* *Volume*** | FRS*Sex*Age | 0.44 | 0.6435 | 2 | 531 |
| ***Hippocampus*** | FRS*Sex*Age | 1.38 | 0.2522 | 2 | 531 |
| ***Log Lateral Ventricle*** | FRS*Sex*Age | 0.83 | 0.4372 | 2 | 531 |
| ***Log WMH*** | FRS*Sex*Age | 0.44 | 0.6427 | 2 | 531 |
| ***Combined Gray*** | FRS*Sex*Age | 1.33 | 0.2666 | 2 | 531 |
| ***Frontal Cortical Gray*** | FRS*Sex*Age | 0.7 | 0.495 | 2 | 531 |
| ***Occipital Cortical Gray*** | FRS*Sex*Age | 4.11 | 0.017 | 2 | 531 |
| ***Temporal Cortical Gray*** | FRS*Sex*Age | 1.75 | 0.1749 | 2 | 531 |
| ***Parietal Cortical Gray*** | FRS*Sex*Age | 0.18 | 0.8364 | 2 | 531 |

Notes:

Models adjusted for Hispanic/Latino heritage group.

Abbreviations: df = degrees of freedom; FRS = Framingham Risk Score; WMH = white matter hyperintensity

Supplemental Table 4. Regression models including main effects and sex and age (3-way) interactions with baseline Framingham Cardiovascular Risk Scores and infarcts, independently.

|  | **Total Brain Volume** | **Hippocampus** | **Log Lateral Ventricle** | **Log WMH** | **Combined Gray** | **Frontal Cortical Gray** | **Occipital Cortical Gray** | **Temporal Cortical Gray** | **Parietal Cortical Gray** |
| --- | --- | --- | --- | --- | --- | --- | --- | --- | --- |
|  | b/ci95 | b/ci95 | b/ci95 | b/ci95 | b/ci95 | b/ci95 | b/ci95 | b/ci95 | b/ci95 |
| Low FRS | ref | ref | ref | ref | ref | ref | ref | ref | ref |
| Medium FRS | 1.44 [-0.12;3.00] | 2.32* [0.43;4.21] | -0.48 [-2.07;1.11] | 0.43 [-0.96;1.82] | 0.24 [-1.29;1.77] | -0.22 [-1.65;1.20] | 0.89 [-0.64;2.43] | 0.24 [-1.46;1.94] | 0.12 [-1.92;2.16] |
| High FRS | 0.50 [-1.34;2.35] | 1.54 [-1.43;4.51] | -2.11 [-4.23;0.01] | 0.86 [-1.37;3.10] | 0.15 [-2.01;2.31] | 0.60 [-1.44;2.64] | -0.68 [-3.17;1.82] | -1.50 [-4.21;1.20] | 1.77 [-0.76;4.30] |
| Female | ref | ref | ref | ref | ref | ref | ref | ref | ref |
| Male | 0.07 [-2.20;2.34] | 3.47** [1.10;5.84] | -1.23 [-3.89;1.43] | -0.16 [-2.21;1.88] | -1.10 [-4.36;2.17] | -2.12 [-5.29;1.06] | 3.32* [0.35;6.29] | -1.57 [-4.51;1.37] | -1.48 [-4.59;1.62] |
| Age | -0.05*** [-0.06;-0.04] | -0.00 [-0.02;0.01] | 0.04*** [0.03;0.05] | 0.04*** [0.03;0.06] | -0.03*** [-0.04;-0.02] | -0.02*** [-0.03;-0.01] | -0.01 [-0.03;0.00] | -0.04*** [-0.05;-0.03] | -0.02* [-0.03;-0.00] |
| Female*Age | ref | ref | ref | ref | ref | ref | ref | ref | ref |
| Male*Age | -0.00 [-0.04;0.04] | -0.06** [-0.10;-0.02] | 0.02 [-0.03;0.07] | 0.00 [-0.03;0.04] | 0.02 [-0.04;0.07] | 0.03 [-0.02;0.09] | -0.05* [-0.11;-0.00] | 0.03 [-0.02;0.08] | 0.02 [-0.03;0.07] |
| Low FRS*Age | ref | ref | ref | ref | ref | ref | ref | ref | ref |
| Medium FRS*Age | -0.02 [-0.05;0.00] | -0.03* [-0.06;-0.01] | 0.01 [-0.01;0.03] | -0.00 [-0.02;0.02] | -0.01 [-0.03;0.02] | 0.00 [-0.02;0.02] | -0.01 [-0.04;0.01] | -0.00 [-0.03;0.02] | -0.01 [-0.04;0.03] |
| High FRS*Age | -0.01 [-0.04;0.01] | -0.02 [-0.06;0.02] | 0.03* [0.00;0.06] | -0.01 [-0.04;0.02] | -0.00 [-0.04;0.03] | -0.01 [-0.04;0.02] | 0.01 [-0.03;0.04] | 0.02 [-0.02;0.06] | -0.03 [-0.06;0.01] |
| Low FRS*Female | ref | ref | ref | ref | ref | ref | ref | ref | ref |
| Low FRS*Male | ref | ref | ref | ref | ref | ref | ref | ref | ref |
| Medium FRS*Female | ref | ref | ref | ref | ref | ref | ref | ref | ref |
| Medium FRS*Male | 0.00 [-2.88;2.89] | -2.59 [-6.74;1.56] | 2.28 [-1.17;5.74] | 0.87 [-1.61;3.35] | -0.67 [-4.49;3.15] | -0.38 [-4.21;3.46] | -5.44** [-9.35;-1.54] | 2.00 [-1.51;5.52] | 1.23 [-2.70;5.16] |
| High FRS*Female | ref | ref | ref | ref | ref | ref | ref | ref | ref |
| High FRS*Male | 1.26 [-2.01;4.52] | -3.14 [-7.78;1.50] | 2.65 [-1.24;6.54] | 0.07 [-3.33;3.48] | 1.55 [-2.61;5.71] | 1.35 [-2.79;5.49] | -1.76 [-6.12;2.61] | 4.13 [-0.37;8.64] | 0.11 [-4.41;4.63] |
| Low FRS*Female*Age | ref | ref | ref | ref | ref | ref | ref | ref | ref |
| Low FRS*Male*Age | ref | ref | ref | ref | ref | ref | ref | ref | ref |
| Medium FRS*Female*Age | ref | ref | ref | ref | ref | ref | ref | ref | ref |
| Medium FRS*Male*Age | -0.01 [-0.05;0.04] | 0.04 [-0.02;0.11] | -0.03 [-0.09;0.02] | -0.02 [-0.06;0.02] | 0.01 [-0.05;0.07] | 0.00 [-0.06;0.06] | 0.09** [0.02;0.15] | -0.03 [-0.09;0.02] | -0.02 [-0.08;0.04] |
| High FRS*Female*Age | ref | ref | ref | ref | ref | ref | ref | ref | ref |
| High FRS*Male*Age | -0.02 [-0.07;0.03] | 0.05 [-0.02;0.12] | -0.04 [-0.10;0.03] | -0.00 [-0.05;0.05] | -0.03 [-0.09;0.04] | -0.03 [-0.09;0.04] | 0.03 [-0.03;0.10] | -0.07 [-0.14;0.00] | -0.01 [-0.08;0.06] |
| Intercept | 3.53*** [2.93;4.13] | 0.14 [-0.95;1.23] | -2.81*** [-3.47;-2.14] | -2.53*** [-3.27;-1.79] | 2.83*** [2.09;3.58] | 2.24*** [1.58;2.89] | 1.38** [0.46;2.30] | 2.63*** [1.97;3.29] | 1.74*** [0.75;2.73] |

|  | **Total Brain Volume** | **Hippocampus** | **Log Lateral Ventricle** | **Log WMH** | **Combined Gray** | **Frontal Cortical Gray** | **Occipital Cortical Gray** | **Temporal Cortical Gray** | **Parietal Cortical Gray** |
| --- | --- | --- | --- | --- | --- | --- | --- | --- | --- |
|  | b/ci95 | b/ci95 | b/ci95 | b/ci95 | b/ci95 | b/ci95 | b/ci95 | b/ci95 | b/ci95 |
| No Infarct | ref | ref | ref | ref | ref | ref | ref | ref | ref |
| MRI Infarct | -0.81 [-2.21;0.59] | -0.39 [-3.52;2.74] | 1.67 [-0.89;4.24] | 1.88* [0.00;3.76] | -0.32 [-1.94;1.31] | -0.02 [-1.87;1.82] | -0.59 [-2.86;1.68] | 0.57 [-1.35;2.50] | -1.15 [-3.14;0.84] |
| Female | ref | ref | ref | ref | ref | ref | ref | ref | ref |
| Male | 1.19* [0.10;2.29] | 1.88* [0.00;3.76] | -0.53 [-2.08;1.03] | 0.63 [-0.37;1.63] | -0.29 [-1.62;1.03] | -1.43* [-2.67;-0.19] | 0.27 [-1.54;2.07] | 0.92 [-0.62;2.46] | 0.22 [-1.31;1.74] |
| Age | -0.06*** [-0.07;-0.05] | -0.01* [-0.03;-0.00] | 0.05*** [0.04;0.06] | 0.05*** [0.04;0.06] | -0.04*** [-0.05;-0.03] | -0.03*** [-0.04;-0.02] | -0.02*** [-0.03;-0.01] | -0.03*** [-0.04;-0.02] | -0.03*** [-0.04;-0.01] |
| Female*Age | ref | ref | ref | ref | ref | ref | ref | ref | ref |
| Male*Age | -0.02* [-0.04;-0.00] | -0.03 [-0.06;0.00] | 0.01 [-0.01;0.04] | -0.01 [-0.02;0.01] | 0.00 [-0.02;0.02] | 0.02 [-0.00;0.04] | -0.00 [-0.03;0.03] | -0.01 [-0.04;0.01] | -0.01 [-0.03;0.02] |
| No Infarct*Female | ref | ref | ref | ref | ref | ref | ref | ref | ref |
| No Infarct*Male | ref | ref | ref | ref | ref | ref | ref | ref | ref |
| MRI Infarct*Female | ref | ref | ref | ref | ref | ref | ref | ref | ref |
| MRI Infarct*Male | -0.39 [-3.81;3.03] | -0.92 [-4.79;2.96] | 0.33 [-3.72;4.39] | -0.84 [-3.93;2.24] | 2.18 [-1.25;5.61] | 3.53* [0.07;6.99] | 0.71 [-3.98;5.40] | -0.03 [-3.24;3.17] | 0.78 [-3.22;4.78] |
| No Infarct*Age | ref | ref | ref | ref | ref | ref | ref | ref | ref |
| MRI Infarct*Age | 0.01 [-0.02;0.03] | 0.01 [-0.04;0.05] | -0.02 [-0.05;0.02] | -0.02 [-0.04;0.01] | 0.00 [-0.02;0.02] | -0.00 [-0.03;0.02] | 0.01 [-0.03;0.04] | -0.01 [-0.04;0.02] | 0.01 [-0.02;0.04] |
| No Infarct*Female*Age | ref | ref | ref | ref | ref | ref | ref | ref | ref |
| No Infarct*Male*Age | ref | ref | ref | ref | ref | ref | ref | ref | ref |
| MRI Infarct*Female*Age | ref | ref | ref | ref | ref | ref | ref | ref | ref |
| MRI Infarct*Male*Age | 0.01 [-0.05;0.06] | 0.01 [-0.04;0.07] | -0.01 [-0.07;0.05] | 0.01 [-0.04;0.05] | -0.03 [-0.08;0.02] | -0.05* [-0.10;-0.00] | -0.01 [-0.08;0.06] | 0.00 [-0.05;0.05] | -0.01 [-0.07;0.05] |
| Intercept | 4.35*** [3.80;4.89] | 0.90* [0.13;1.67] | -3.56*** [-4.11;-3.00] | -3.08*** [-3.66;-2.50] | 3.27*** [2.62;3.92] | 2.76*** [2.11;3.41] | 1.72*** [1.11;2.33] | 2.33*** [1.63;3.02] | 2.36*** [1.60;3.12] |

Notes:

Infarct(s) assessed on magnetic resonance imaging scan.

Main effects from interaction models have limited interpretability

Models adjusted for Hispanic/Latino heritage group.

Abbreviations: b = beta; ci = confidence interval; FRS = Framingham Risk Score; WMH = white matter hyperintensity

Supplemental Table 5. Wald (F)-tests for main effects and three-way sex and age interactions with infarcts.

|  | **MRI Infarcts** | | | | |
| --- | --- | --- | --- | --- | --- |
| **Brain Outcome** | **Exposure** | **F-Test** | **p-value** | **df1** | **df2** |
| ***Total Brain* *Volume*** | Infarcts*Sex*Age | 0.04 | 0.8475 | 1 | 532 |
| ***Hippocampus*** | Infarcts*Sex*Age | 0.2 | 0.6537 | 1 | 532 |
| ***Log Lateral Ventricle*** | Infarcts*Sex*Age | 0.11 | 0.7363 | 1 | 532 |
| ***Log WMH*** | Infarcts*Sex*Age | 0.12 | 0.7249 | 1 | 532 |
| ***Combined Gray*** | Infarcts*Sex*Age | 1.64 | 0.2012 | 1 | 532 |
| ***Frontal Cortical Gray*** | Infarcts*Sex*Age | 4.42 | 0.0359 | 1 | 532 |
| ***Occipital Cortical Gray*** | Infarcts*Sex*Age | 0.11 | 0.7438 | 1 | 532 |
| ***Temporal Cortical Gray*** | Infarcts*Sex*Age | 0 | 0.9618 | 1 | 532 |
| ***Parietal Cortical Gray*** | Infarcts*Sex*Age | 0.13 | 0.7212 | 1 | 532 |

Notes:

Infarct(s) assessed on magnetic resonance imaging scan.

Models adjusted for Hispanic/Latino heritage group.

Abbreviations: df = degrees of freedom; MRI = magnetic resonance imaging; WMH = white matter hyperintensity

Supplemental Figure 1. Sex specific, over age (years), associations between infarcts with frontal cortical gray matter volumes in the Study of Latinos-Investigation of Neurocognitive Aging Magnetic Resonance Imaging (SOL-INCA MRI)

Notes:

Infarct(s) assessed on magnetic resonance imaging scan.

Estimates derived from models that included main effects for sex, age, and Hispanic/Latino heritage groups and three-way interactions between sex, age, and infarcts.

Abbreviation: Std = Standardized residualized for cranial size.

Supplemental Figure 2. Pairwise comparisons of heritage groups on white matter hyperintensity volumes.


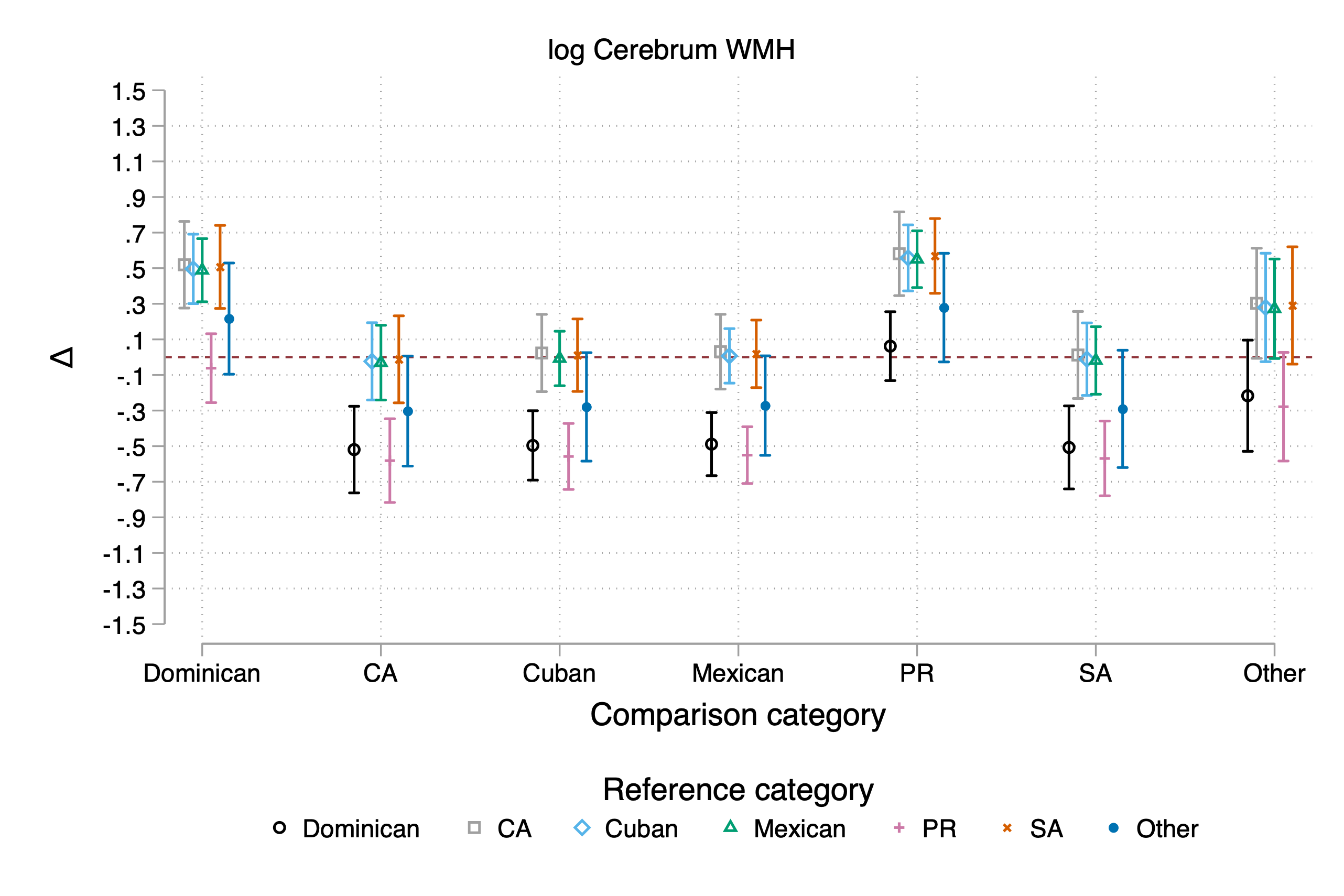


Notes:

Estimates derived from models that included main effects for sex, age, and cardiovascular disease risk. Adjusting for infarcts in place of cardiovascular disease risk did not change the overall pattern of results.

Abbreviation: CA = Central American; PR = Puerto Rican; SA = South American; WMH = white matter hyperintensity

Supplemental Figure 3. Pairwise comparisons of heritage groups on cortical gray matter volumes.


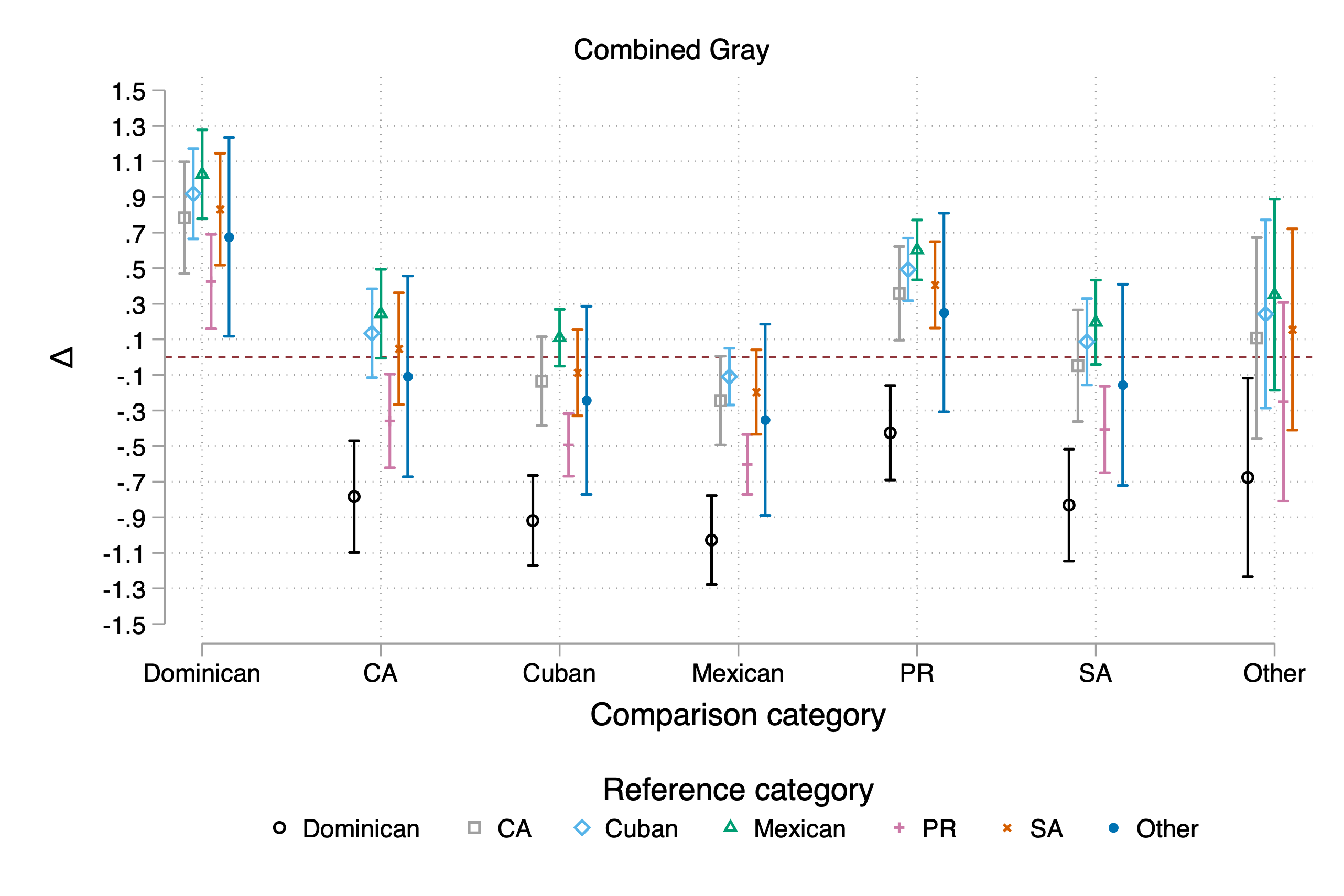


Notes:

Estimates derived from models that included main effects for sex, age, and cardiovascular disease risk. Adjusting for infarcts in place of cardiovascular disease risk did not change the overall pattern of results.

Abbreviation: CA = Central American; PR = Puerto Rican; SA = South American

Supplemental Figure 4. Pairwise comparisons of heritage groups on frontal gray matter volumes.


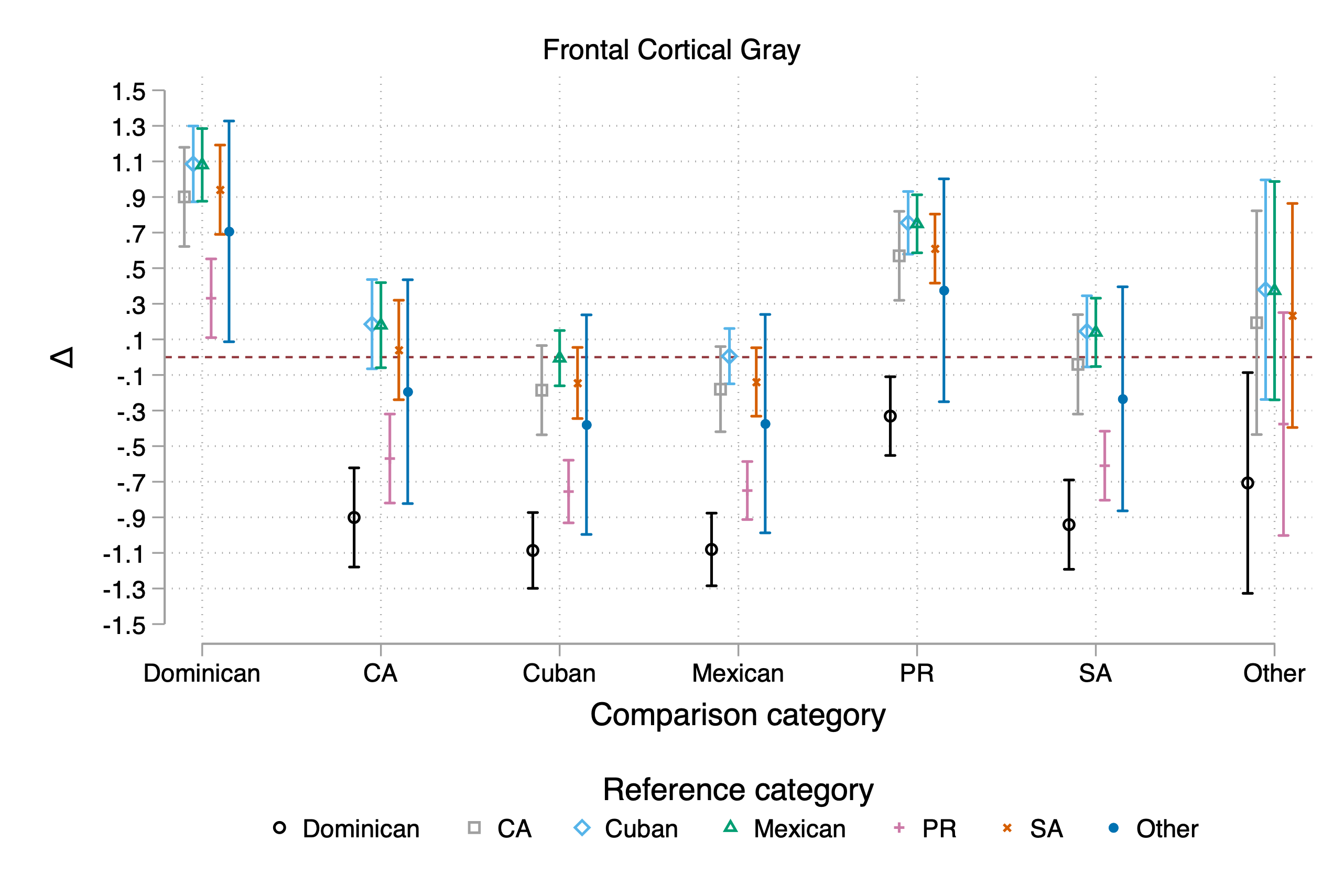


Notes:

Estimates derived from models that included main effects for sex, age, and cardiovascular disease risk. Adjusting for infarcts in place of cardiovascular disease risk did not change the overall pattern of results.

Abbreviation: CA = Central American; PR = Puerto Rican; SA = South American

Supplemental Figure 5. Pairwise comparisons of heritage groups on occipital gray matter volumes.


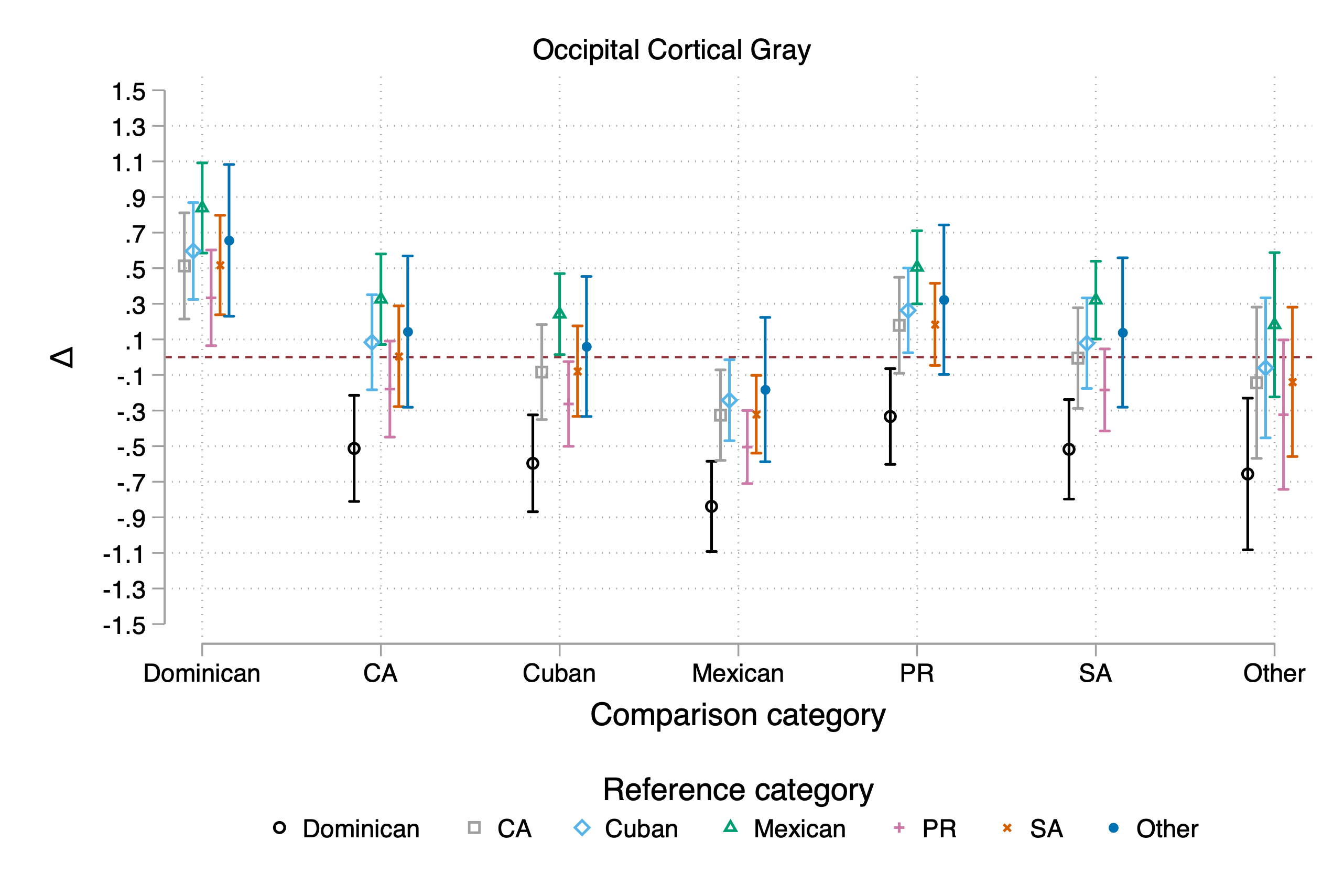


Notes:

Estimates derived from models that included main effects for sex, age, and cardiovascular disease risk. Adjusting for infarcts in place of cardiovascular disease risk did not change the overall pattern of results.

Abbreviation: CA = Central American; PR = Puerto Rican; SA = South American

Supplemental Figure 6. Pairwise comparisons of heritage groups on parietal gray matter volumes


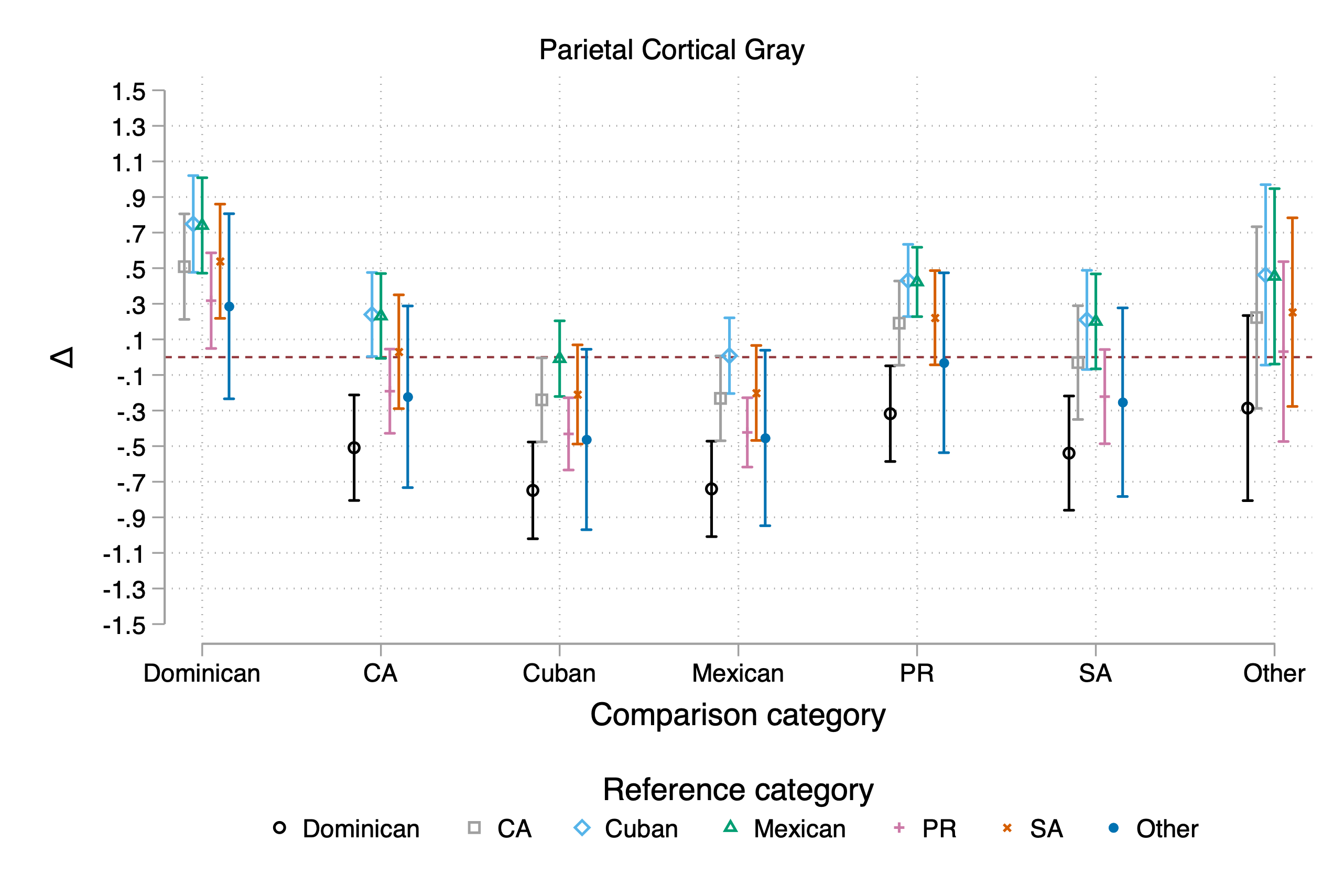


Notes:

Estimates derived from models that included main effects for sex, age, and cardiovascular disease risk. Adjusting for infarcts in place of cardiovascular disease risk did not change the overall pattern of results.

Abbreviation: CA = Central American; PR = Puerto Rican; SA = South American
